# Sex dimorphism of IL-17-secreting peripheral blood mononuclear cells in ankylosing spondylitis based on bioinformatics analysis and machine learning

**DOI:** 10.1186/s12891-024-07589-6

**Published:** 2024-06-24

**Authors:** Sifang Li, Hua Chao, Zihao Li, Siwen Chen, Jingyu Zhang, Wenjun Hao, Shuai Zhang, Caijun Liu, Hui Liu

**Affiliations:** 1 Department of Spine Surgery, The First Affiliated Hospital, Sun Yat-sen University, No.58 Zhongshan 2nd Road, Guangzhou, Guangdong 510080 China; 2 Guangdong Province Key Laboratory of Orthopaedics and Traumatology, Guangzhou, Guangdong 510080 China; 3 Department of Spine Surgery, The Third Affiliated Hospital of Guangzhou University of Traditional Chinese Medicine, Guangzhou, 510378 China; 4 Guangdong research institute for Orthopedics & Traumatology of Chinese Medicine, No. 22, Qingzhu Street, Jiangnan West Road, Guangzhou, 510378 China

**Keywords:** Sex dimorphism, Interleukin-17, Ankylosing spondylitis, Machine learning, Bioinformatics analysis

## Abstract

**Background:**

Ankylosing spondylitis (AS) with radiographic damage is more prevalent in men than in women. IL-17, which is mainly secreted from peripheral blood mononuclear cells (PBMCs), plays an important role in the development of AS. Its expression is different between male and female. However, it is still unclear whether sex dimorphism of IL-17 contribute to sex differences in AS.

**Methods:**

GSE221786, GSE73754, GSE25101, GSE181364 and GSE205812 datasets were collected from the Gene Expression Omnibus (GEO) database. Differential expressed genes (DEGs) were analyzed with the Gene Set Enrichment Analysis (GSEA), Gene Ontology (GO) and Kyoto Encyclopedia of Genes and Genomes (KEGG) methods. CIBERSORTx and EcoTyper algorithms were used for immune infiltration analyses. Machine learning based on the XGBoost algorithm model was used to identify the impact of DEGs. The Connectivity Map (CMAP) database was used as a drug discovery tool for exploring potential drugs based on the DEGs.

**Results:**

According to immune infiltration analyses, T cells accounted for the largest proportion of IL-17-secreting PBMCs, and KEGG analyses suggested an enhanced activation of mast cells among male AS patients, whereas the expression of TNF was higher in female AS patients. Other signaling pathways, including those involving metastasis-associated 1 family member 3 (MAT3) or proteasome, were found to be more activated in male AS patients. Regarding metabolic patterns, oxidative phosphorylation pathways and lipid oxidation were significantly upregulated in male AS patients. In XGBoost algorithm model, DEGs including METRN and TMC4 played important roles in the disease process. we integrated the CMAP database for systematic analyses of polypharmacology and drug repurposing, which indicated that atorvastatin, famciclocir, ATN-161 and taselisib may be applicable to the treatment of AS.

**Conclusions:**

We analyzed the sex dimorphism of IL-17-secreting PBMCs in AS. The results showed that mast cell activation was stronger in males, while the expression of TNF was higher in females. In addition, through machine learning and the CMAP database, we found that genes such as METRN and TMC4 may promote the development of AS, and drugs such as atorvastatin potentially could be used for AS treatment.

**Supplementary Information:**

The online version contains supplementary material available at 10.1186/s12891-024-07589-6.

## Introduction

Ankylosing spondylitis (AS), a subtype of axial spondyloarthritis (SpA), is characterized by chronic back pain and the formation of syndesmophytes, which may lead to spinal fusion or ankylosis [1]. The prevalence of AS in an east Asian population was reported to be 0.79% [2]. Approximately two-thirds of actively employed individuals with AS have work-related issues, leading to substantial direct and indirect costs to society [3].

Clinical observations have highlighted that sex plays a pivotal role in the manifestation of AS, which is unique among SpA subtypes [4]. Unlike most SpA variants, AS exhibits a pronounced male predominance, with a male-to-female ratio of up to 3:1 [4, 5]. In addition, several factors associated with the disease differ significantly between male and female patients. Female patients tend to have a higher Bath Ankylosing Spondylitis Functionality Index (BASFI) [6–8] and are less responsive to treatment with TNF-inhibitors [9]. Female patients also tend to have a lower modified Stoke Ankylosing Spondylitis Spine Score (mSASSS) [10, 11] and lower C-reactive protein levels [8] compared with male patients, which means that female AS patients tend to have a lower degree of inflammation and slower imaging progression These observations implicate a sex bias in the immunopathogenesis of AS.

IL-17-secreting cells, which producing IL17, contribute to the pathogenesis of AS [12]. The role of IL-17 in AS is currently believed to be linked to inflammation and to neutrophil activity through its induction of the production of IL-6 and IL-8 [13], as well as by enhancing osteoclastogenesis via receptor activator of NF-κB ligand (RANKL) pathway [14]. Notably, these IL-17-secreting cells also expressed TNF [15], which appears to be key in the inflammatory response observed in AS [16]. Additional research has demonstrated that IL-17-secreting cells express more effector molecules, including proinflammatory cytokines and chemokines such as CXCL3, CCL4, CCL5, IL-3, and IL-22 [17]. Collectively, this evidence suggests that, beyond IL-17, these cells secrete a spectrum of other proteins that may contribute to the exacerbation of AS.

Many literatures have shown that sex has an effect on the functions of IL17-secreting cells. Studies have indicated that PPARγ-deficient T cells in females are more easily activated and differentiate into IL-17-secreting cells [18]. In the ovariectomized mouse model, it was observed that absence of estrogen resulted in the proliferation of IL-17-secreting cells [19]. Clinical observations have also revealed that female patients exhibit elevated levels of IL-17 expression during urinary tract infections compared to their male counterparts [20]. As IL-17-secreting cells is a key factor in AS, it is possible that the differential expression of IL-17-secreting cells determines the difference in disease susceptibility and severity between male and female patients. Consequently, we thought that comparing the expression profiles of IL-17-secreting cells would provide a clearer understanding of the key factors for male susceptibility and potential therapeutic targets.

In this study, we used functional enrichment analysis, immune infiltration analysis and machine learning to analyze the gene expression profiles of IL-17-secreting PBMCs from male and female AS patients. we observed that the activation of mast cells was stronger in male patients, while TNF signaling pathway was more activated in female AS patients regarding PBMC metabolic patterns, we found that oxidative phosphorylation pathways and lipid oxidation were significantly upregulated in male patients. Finally, we identified multiple such DEGs, including METRN and TMC4, that may account for the higher incidence of AS in males.

## Methods

### Data collection

AS datasets were obtained from the public repository NCBI GEO using “ankylosing spondylitis” as the search query. The target species was set to “human,” and the entry type was “series.” A total of 38 datasets were retrieved in this way. In order to ensure the quality of the data, we checked each of these datasets for information related to the experimental sample, experimental design and data type. Ultimately, five datasets (GSE221786, GSE73754, GSE25101, GSE181364 and GSE205812) were identified from the GEO database.

GSE221786 included data from RNA-seq experiments performed on platform GPL24676. It contained gene expression profiles from whole blood samples that included isolated PBMCs from control and AS patients that had been stimulated with CytoSim for 4 h to enrich the cells in IL-17. These samples were from 20 AS patients (11 male and 9 female) and 8 healthy controls (4 male and 4 female). GSE73754 (array, platform GPL10558) included gene expression profiles of whole blood samples from 52 AS patients and 20 healthy controls. GSE25101 (array, platform GPL6947) included gene expression profiles of whole blood samples from 16 AS patients and 16 healthy controls. GSE181364 (RNA-seq, platform GPL24676) included gene expression profiles of whole blood samples from 5 AS patients and 3 healthy controls. GSE205812 (RNA-seq, platform GPL24676) included gene expression profiles of whole blood samples from 3 AS patients and 3 healthy controls. Data from GSE221786 were used to conduct the main analysis of this article. Datasets GSE221786, GSE73754, GSE25101, GSE181364 and GSE205812 were used to train XGBoost machine learning models [21]. All gene expression information obtained from the database are available in supplementary file 1. GSE181364 was approved by the Ethics Committee of Anhui Medical University (No. 20,200,740). GSE205812 was approved and reviewed by the Medical Ethics Review Committee of the Affiliated Hospital of Qingdao University (approval number: QYFY WZLL 27,251). Our manuscript has been approved by the First Affiliated Hospital of Sun Yat-sen University with the approval number: No. 2,024,052,502.

### Screening for differentially expressed genes

A standardized matrix file of the microarray data was provided with dataset GSE221786. The “limma” package of R was used to identify DEGs between 20 AS samples and 8 healthy controls in GSE73754 with inclusion criteria of *p* < 0.05 and |logFC| > 0. The expression levels of DEGs between AS subjects and healthy controls were visualized using the “ggplot2” and “pheatmap” R packages. Significant correlations between DEGs were visualized using the “corrplot” and “circlize” packages. Volcano plots were developed and Principal Component Analyses (PCA) were performed using the OmicShare tools (https://www.omicshare.com/tools) with the default settings. A heatmap was plotted using an online platform for data analysis and visualization (https://www.bioinformatics.com.cn).

### Functional enrichment analysis

Gene Set Enrichment Analysis (GSEA), Gene Ontology (GO) and Kyoto Encyclopedia of Genes and Genomes (KEGG) are commonly used methods to perform gene enrichment analyses. GSEA was performed to identify differential pathways between male and female subjects. Pre-ranked gene lists were generated based on the correlation of gene expression with the treatment condition using R package “clusterProfiler“ [22], with “c2.cp.v7.2.symbols.gmt” (https://www.gsea-msigdb.org/gsea/msigdb/index.jsp). Gene sets from the Molecular Signatures Database (MSigDB) were utilized for enrichment analyses, which were conducted using GSEA software (Broad Institute, Cambridge, MA, USA) with 200 permutations to estimate false discovery rates (FDR). The gene sets were considered significantly enriched at FDR < 0.25 and *p* < 0.05. The GSEA results were visualized by enrichment plots, highlighting key pathways that were significantly enriched in the condition of interest. To understand the biological functions of genes, GO terms and KEGG pathway analyses were applied for taxonomy-based analysis with the “ClusterProfiler” R package [22, 23]. In addition, to illustrate the differences in genes involved in oxidative phosphorylation, we used the signal pathway map inherent in KEGG [24].

### Immune infiltration analysis

A deconvolution approach analysis algorithms were used for immune infiltration analyses [25]. The deconvolution approach can be an effective solution to discern specific immune cell type proportions from transcriptomic data of heterogeneous samples. Specifically, the deconvolution approac have generated an RNA-sequencing (RNA-seq) gene expression profile encompassing immune cells that constitute the PBMCs fraction. This comprehensive profiling was complemented with data obtained from fluorescence-activated cell sorting (FACS) to ascertain the proportions of these immune cells, along with an assessment of gene expression levels within the PBMCs.

Correlations between DEGs and immune cell infiltration in AS were also analyzed. For this analysis, data from healthy controls were omitted, and data representing expression of DEGs was extracted. Correlation analyses were performed with the results of immune infiltration according to Pearson’s correlation coefficient. The results of these analyses were presented using the “ggplot2” package.

### Machine learning

In the model-development phase, an XGBoost algorithm model [21] was constructed to analyze the contribution of DEGs between AS patients and healthy controls. A backward stepwise analysis was performed to select variables with a threshold of *p* < 0.05 according to the Akaike information criterion.

### The prediction of potential small-molecule drugs

The Connectivity Map (CMAP) database (https://clue.io/) is a drug discovery tool for exploring potential biological associations among diseases, genes and drugs. Specifically, the model allows the prediction of small molecule drugs that may induce or reverse the biological processes associated with DEGs. In this study, drugs with negative scores in T cell-related cell lines (for example, Jurkat cells) and osteogenesis-related cell lines (for example, U2OS cells) were explored, and information regarding the clinical research stages of the drugs and their mechanisms of action were drawn from information provided within the database.

## Results

### Sex dimorphism of immune cells cell among patients

**Fig. 1:**
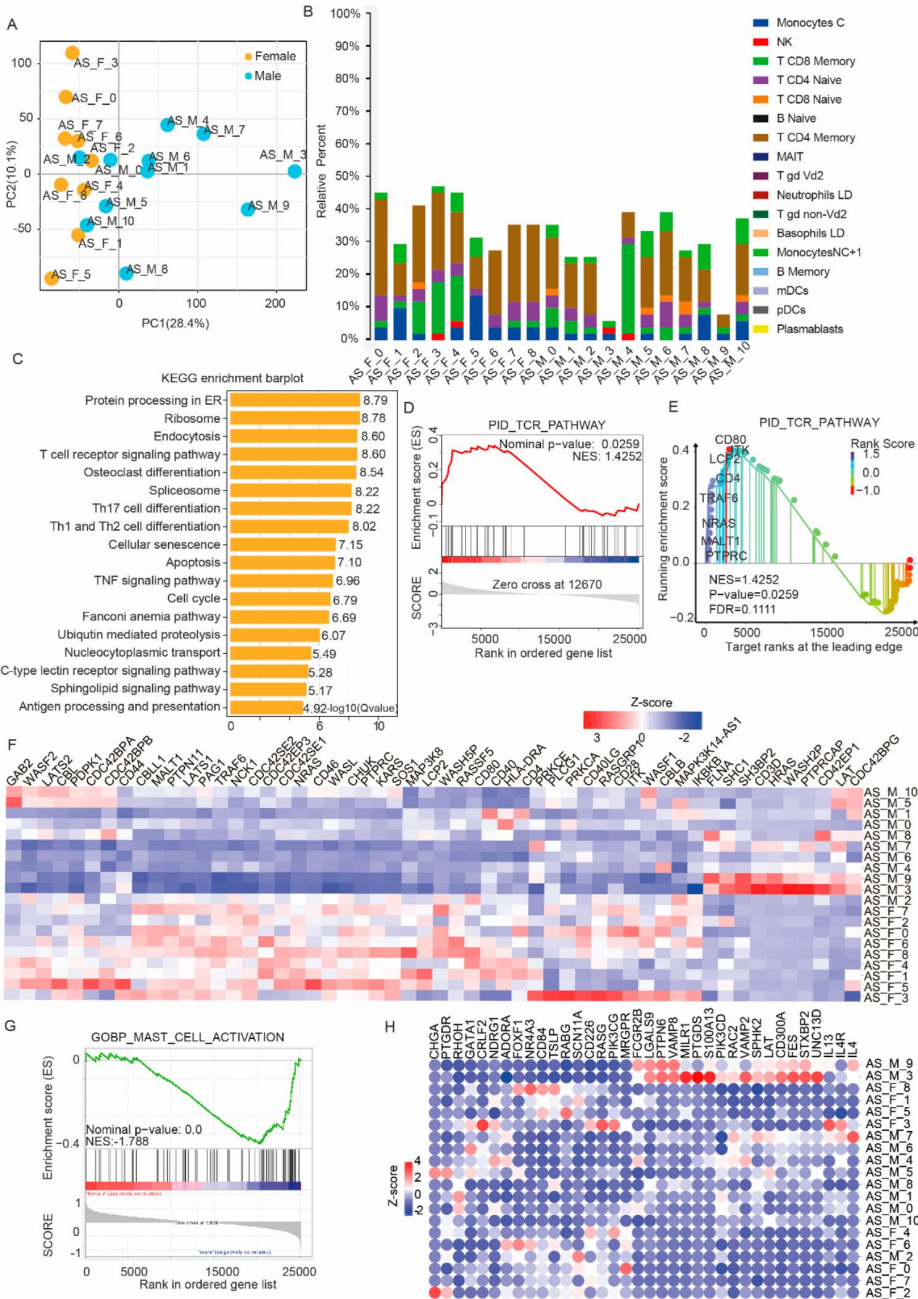
Immune infiltration and enrichment analyses of immune-related signaling pathways between male and female subjects with ankylosing spondylitis (AS). **A** A Principal Component Analysis (PCA) showing differences in gene expression between male and female individuals with AS. **B** A deconvolution approach analysis showing sex differences in immune infiltration among individuals with AS. Bar plot shows relative composition of PBMCs. **C** KEGG pathway enrichment analysis of differentially expressed genes (DEGs) between male and female AS patients. **D**, **E** GSEA enrichment plot **D** and diagram **E** of IL-17-secreting cells from male patients with AS compared with IL-17-secreting cells from female patients with AS focusing on genes involved in T-cell receptor (TCR) signaling. **F** Heatmap showing the DEGs related to the PID_TCR_PATHWAY dataset between male and female patients with AS. **G** GSEA enrichment plots of IL-17-secreting cells from male patients with AS compared with IL-17-secreting cells from female patients with AS from the GOBP_MAST_CELL_ACTIVATION dataset. H) Heatmap showing the DEGs related to the GOBP_MAST_CELL_ACTIVATION dataset between male and female AS patients

In database GSE221786, we obtained RNA-seq data from cells secreting IL-17 PBMC from 11 male and 9 female AS patients. We first observed the overall gene expression differences between male and female patients and found significant differences (Fig. 1A). Next, we used a deconvolution approach to investigate the sequencing results for information regarding immune cell infiltration [25], and we found that CD4 + T cell accounted for the majority of these IL-17 secreting cells, followed by CD8 + T cells (Fig. 1B). However, it is essential to acknowledge that deconvolution approach is an analytical tool designed to infer gene expression profiles and estimate the relative abundance of constituent cell types within a heterogeneous cell population. we have observed that the sum of the proportions of the recognized cell types does not amount to 100%. This discrepancy suggests that there may be other types of PBMCs that are responsible for the secretion of IL-17, which were not initially accounted for in our analysis.

We then used a KEGG analysis to uncover differences in the activation of cellular signaling pathways. We found that among the immune-related pathways, T-cell receptor (TCR)-related genes were the most significantly enriched (Fig. 1C). In addition, according to GSEA, the activation of the TCR signaling pathway was more prominent in female subjects than in male subjects (Fig. 1D, E). Specifically, the upregulation of expression of PTPRC suggests a stronger T cell activation in female AS patients as compared to the activation in male AS patients (Fig. 1F). A GSEA enrichment analysis showed that among IL17-secreting cells from male subjects, the activation of mast cells was more prominent as compared to female subjects (Fig. 1G). We identified multiple upregulated genes associated with mast cell activation in male subjects, including IL13, PTPN6, VAMP8 and LAT2 (Fig. 1H).

### Immune infiltration and enrichment analyses of immune-related signaling pathways between normal male and female subjects

In a comparative analysis of the patients, it was observed that male individuals displayed a stronger activation of mast cells, accompanied by elevated expression of key genes such as LAT2. We further investigated whether this difference was generally associated with sex or represented a specific manifestation within the context of AS disease. Therefore, we compared immune infiltration and related signaling pathways between normal male and female subjects.

**Fig. 2:**
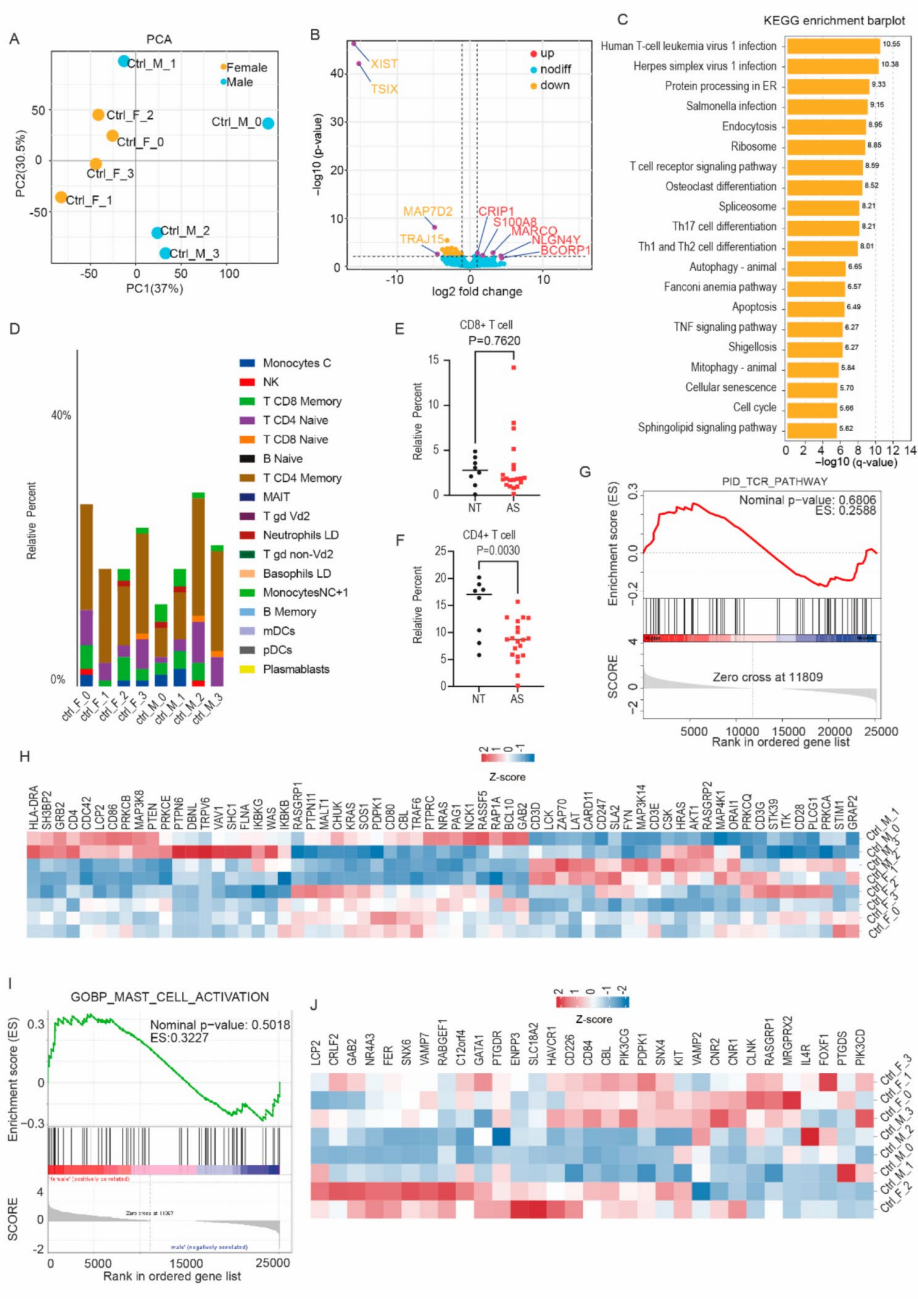
Immune infiltration and enrichment analyses of immune-related signaling pathways between male and female healthy control subjects. **A** A Principal Component Analysis (PCA) showing differences in gene expression between healthy control male and female individuals. **B** A volcano map displaying differentially expressed genes (DEGs) between male and female control subjects. **C** KEGG pathway enrichment analysis of DEGs between male and female control subjects. **D** a deconvolution approach analysis showing differences in immune infiltration between male and female control subjects. **E**, **F** Comparison of the numbers of CD4 + and CD8 + T cells between the ankylosing spondylitis (AS) group (*n* = 20) and the control group (*n* = 8). P-values were obtained with t-tests. **G** GSEA enrichment plots of IL-17-secreting cells from male controls compared with IL-17-secreting cells from female controls with AS using the PID_TCR_PATHWAY dataset. **H** Heatmap showing the DEGs related to the PID_TCR_PATHWAY dataset between male and female healthy control subjects. **I** GSEA enrichment plots of IL-17-secreting cells from male control subjects compared with IL-17-secreting cells from female controls with AS for the GOBP_MAST_CELL_ACTIVATION dataset. **J** Heatmap showing DEGs related to the GOBP_MAST_CELL_ACTIVATION dataset between control male and female individuals

We analyzed overall gene expression in male and female control subjects who had not been diagnosed with AS, and we found significant differences (Fig. 2A). In particular, we found that female subjects had higher average expression levels of XIST, TSIX and other non-coding RNAs located on the X chromosome. In male subjects, we identified several specific overexpressed protein-coding genes on autosomes, including CRIP1, S100A8, MARCO, NLGN4Y and BCORP1 (Fig. 2B). We then performed KEGG analyses of DEGs to investigate the activation of cellular signaling pathways, and we found that among immune-related pathways, genes related to the TCR signaling pathway were the most significantly enriched (Fig. 2C).

With regard to immune infiltration, a deconvolution approach analysis demonstrated that in control subjects, as in patients diagnosed with AS, T cells were the predominant IL-17-secreting cells (Fig. 2D). There was no statistical difference in CD8 + T cell invasion compared to the AS group (Fig. 2E). However, we found that a decrease in the proportion of CD4 + T cells among patients with AS compared to healthy individuals (Fig. 2F).

We performed GSEA focused on the activation of T cells and mast cells in male and female control subjects. We found that there was no difference in the activation of T cells and mast cells between male and female control subjects, despite significant differences in the secretion of IL-17 (Fig. 2G-J).

### Sex-specific pathways in IL-17-secreting cells

**Fig. 3:**
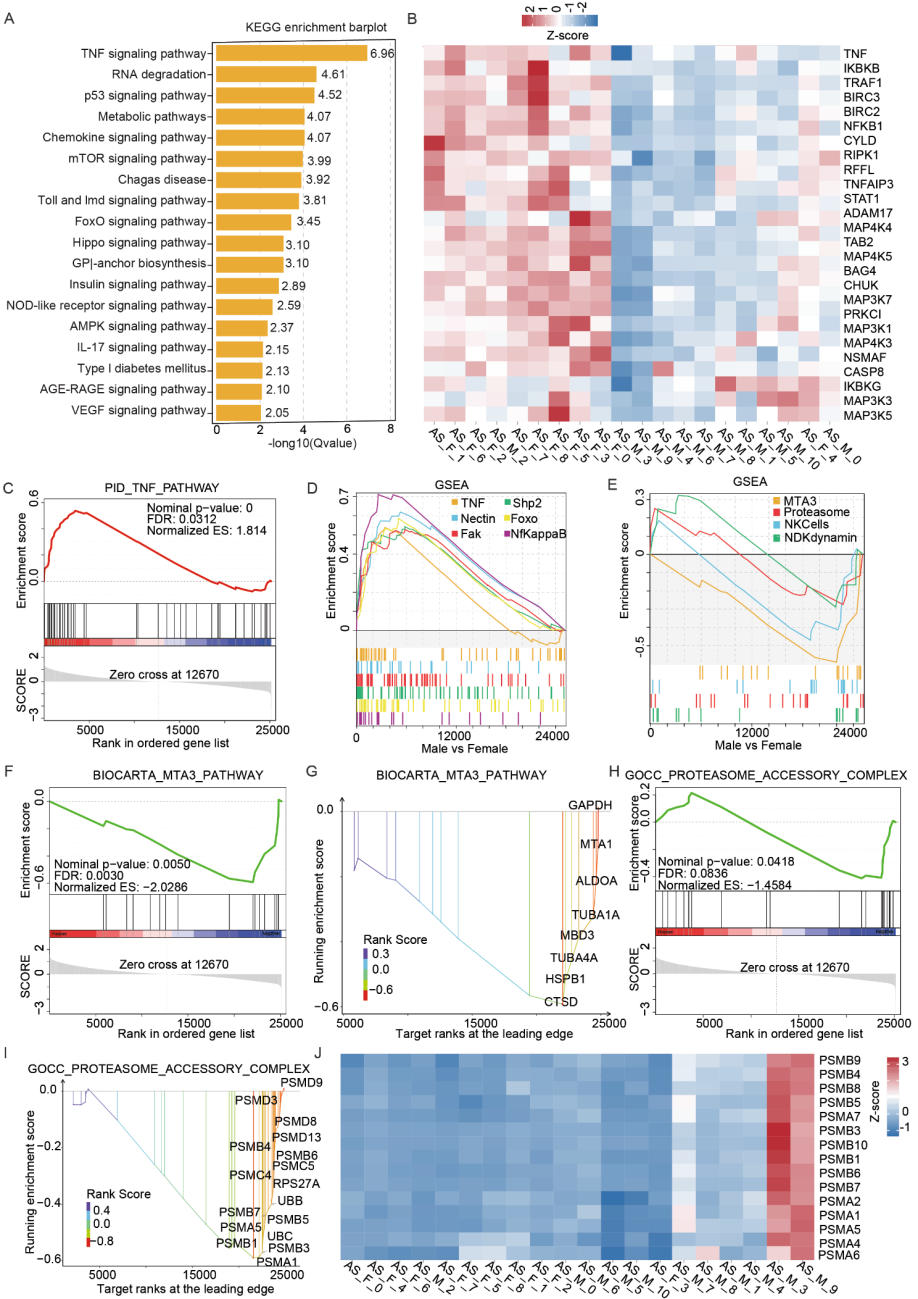
Sex-specific pathways within IL-17-secreting cells. **A** KEGG pathway enrichment analysis of differentially expressed genes (DEGs) related to signal transduction pathways between male and female control subjects. **B** Heatmap showing the DEGs related to the PID_TNF_PATHWAY between male and female individuals with AS. **C** GSEA enrichment plots of IL-17-secreting cells from male patients with AS compared with IL-17-secreting cells from female patients with AS for the PID_TNF_PATHWAY and PID_NECTIN_PATHWAY. **D**, **E** GSEA enrichment plots showing the most highly enriched signaling pathways in female **D** and male **E** subjects. **F**-**I** GSEA enrichment plots and diagrams of IL-17-secreting cells from male patients with AS compared with female patients with AS for BIOCARTA_MTA3_PATHWAY **F**, **G** and GOCC_PROTEASOME_ACCESSORY_COMPLEX **H**, **I**. **J** Heatmap showing the DEGs related to the GOCC_PROTEASOME_ACCESSORY_COMPLEX between male and female individuals with AS

Considering the significant impact of signal pathway activation on cellular outcomes, we analyzed the differences in the expression of genes involved in pathways leading to IL-17 secretion cell activation pathways between male and female AS patients. In the KEGG pathway enrichment analysis on the relevant DEGs, we observed that the TNF signaling pathway was the most enriched (Fig. 3A). Interestingly, TNF signaling is one of the pathways most extensively studied in AS disease [26]. GSEA revealed that the expression of TNF signaling-related genes in samples associated with female subjects was significantly increased (Fig. 3B, C). In particular, the expression level of TNF itself was found to be significantly increased in women (Fig. 3B).

We explored in more depth the signaling pathways that are activated in female AS patients compared to male patients. Our findings revealed that the pathways involving nectin, FAK, SHP2 and FOXO also demonstrated increased activation in female patients at the level of gene transcription (Fig. 3D). However, as the prevalence of AS is higher in male subjects than in female subjects, the signaling pathways activated in males may hold more significance for disease progression. Therefore, we also analyzed the signaling pathways that were more highly activated in male patients and discovered that genes involved in pathways related to MTA3, the proteasome, NKC cells and NDK/dynamin were more highly expressed in male patients compared to female patients (Fig. 3E-J). We proposed that these signaling pathways may represent potential therapeutic targets for the treatment of AS.

### Differences in metabolic patterns of IL-17-secreting cells between male and female AS patients

**Fig. 4:**
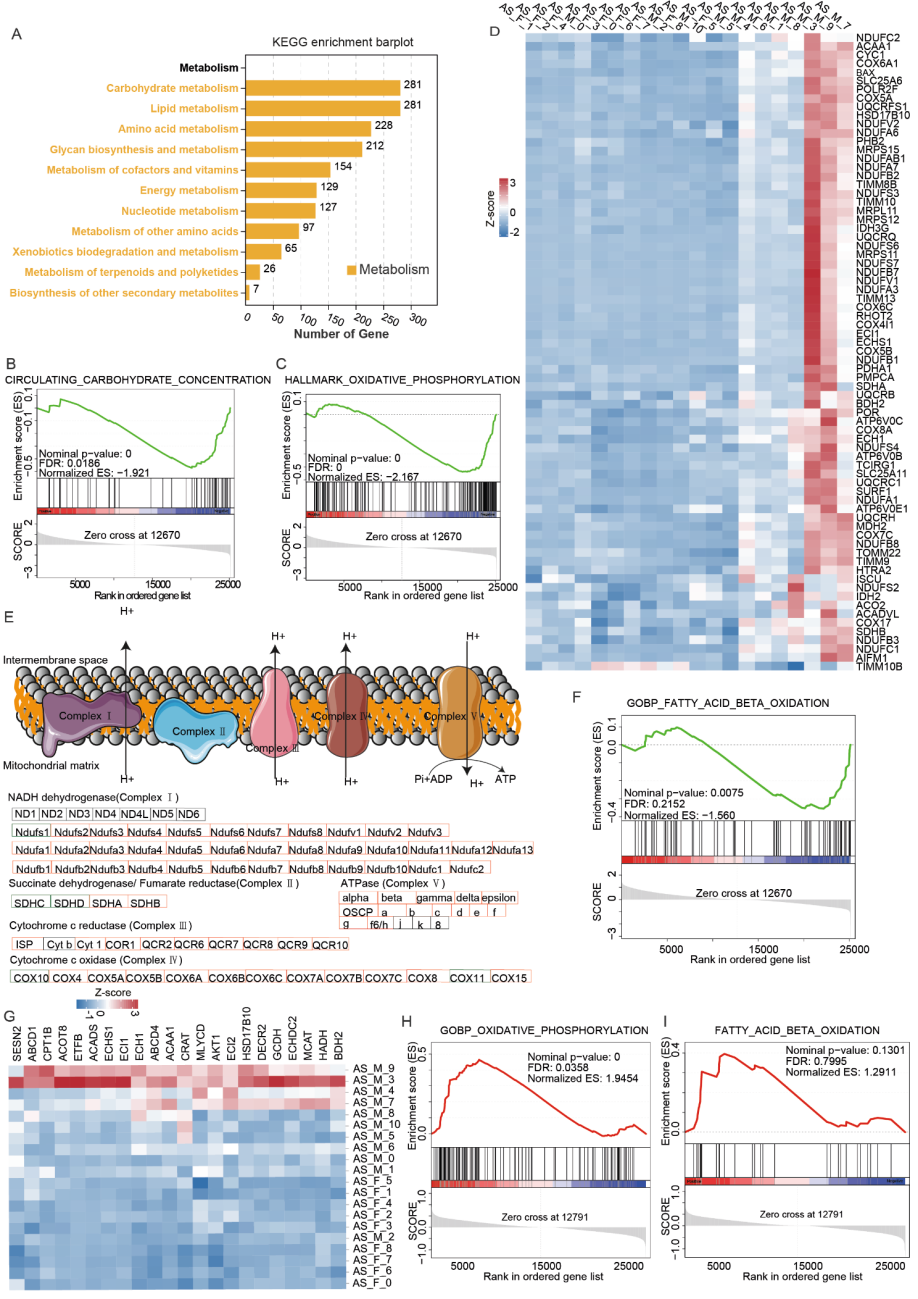
Differences in metabolic patterns of IL-17-secreting cells between male and female ankylosing spondylitis (AS) patients. **A** KEGG pathway enrichment analysis of differentially expressed genes (DEGs) related to metabolism between male and female control subjects. **B**, **C** GSEA enrichment plots and diagram of IL-17-secreting cells from male patients with AS compared with IL-17-secreting cells from female patients with AS for the circulating carbohydrate concentration and hallmark oxidative phosphorylation pathways. **D** Heatmap showing the upregulated DEGs related to the hallmark oxidative phosphorylation dataset between males and females with AS. **E** In the signal pathway diagram of the electronic respiratory chain, the red box represents the genes that are elevated in male patients compared to female patients. The green box indicates the genes that are elevated in female patients compared to male patients. The black box indicates genes that are not changed between male and female patients. **F**, **G** GSEA enrichment plots and diagram of IL-17-secreting cells from male patients with AS compared with IL-17-secreting cells from female patients with AS for the GOBP_fatty_acid_beta_oxidation dataset. **H**, **I** GSEA enrichment plots of IL-17-secreting cells from male patients with AS compared with IL-17-secreting cells from control group of males for GOBP_OXIDATIVE_PHOSPHORYLATION and FATTY_ACID_BETA_OXIDATION datasets

In addition to the signaling pathways mentioned above, based on previous studies showing that the metabolic pattern of immune cells greatly influences their status [27, 28], we analyzed the differences in metabolic patterns of IL-17-secreting cells between male and female subjects. We performed KEGG pathway enrichment analysis on the DEGs between male and female subjects with AS, with particular focus on metabolism-related pathways. We found that carbohydrate metabolism and lipid metabolism were the most enriched pathways (Fig. 4A).

Next, we performed GSEA and found that male subjects exhibited greater enrichment of genes in carbohydrate metabolism and aerobic phosphorylation pathways (Fig. 4B, C). In addition, male AS subjects also exhibited upregulation in genes encoding proteins in oxidative phosphorylation respiratory chain complexes I through V (Fig. 4D, E). GSEA demonstrated that male subjects exhibited an enrichment of upregulated genes in the fatty acid oxidation pathway (Fig. 4F, G). Specific genes upregulated in male AS subjects included PCK2, BDH2, ACADS, ECHDC2, ACAA1, AKT1, ABCD4, CRAT and ECH1 (Fig. 4G). Meanwhile, we also compared the changes in metabolic patterns between male patients with AS and normal male subjects, and we found that the expression levels of genes associated with oxidative phosphorylation were increased in AS patients, but the levels of genes associated with fatty acid oxidation remained unchanged (Fig. 4H, I). These results suggest that changes in oxidative phosphorylation may contribute to the onset of the disease.

### Sexual dimorphism in secretory proteins of IL-17-secreting cells

**Fig. 5:**
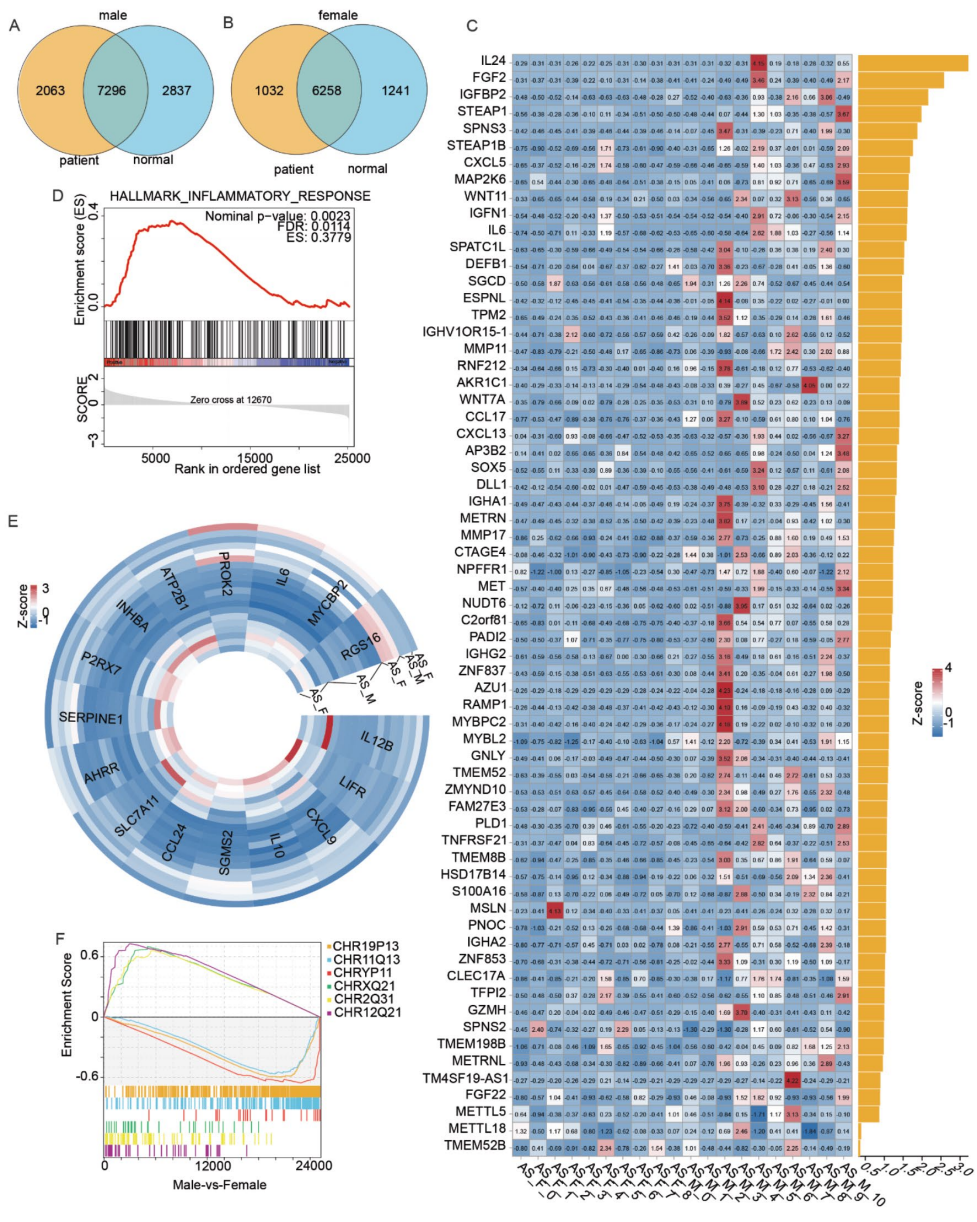
Sexual dimorphism in secretory proteins in IL-17-secreting cells. **A**, **B** Venn diagrams displaying the genes elevated in (A) male and (B) female ankylosing spondylitis (AS) patients compared to female patients, and the genes with elevated transcriptional levels in control subjects compared to female control subjects. **C** Heatmap showing the differentially expressed genes related to secretory proteins. **D**, **E** GSEA enrichment plots and heatmap of IL-17-secreting cells from male patients with AS compared with female patients with AS for the HALLMARK_INFLAMMATORY_RESPONSE dataset. **F** GSEA enrichment plots and diagram of IL-17-secreting cells from male patients with AS compared with female patients with AS according to the chromosomal location of the gene

AS symptoms primarily manifest in localized regions of the spine, and circulating immune cells that act at these locales along with their secreted proteins play important roles in disease progression. Therefore, we initially identified genes that were more highly expressed in male and female patients compared to control groups (Fig. 5A, B), and then we identified genes in these sets that encode secreted proteins. We discovered that a few of the secreted proteins overexpressed at the transcriptional level in male AS patients were not pro-inflammatory molecules (Fig. 5C). However, transcripts encoding proteins promoting osteogenesis, such as FGF2, WNT and DLL1, were found to be highly expressed.

In contrast, the expression of pro-inflammatory molecules, such as IL-6 and CCL24, was higher in female AS patients as compared to male patients (Fig. 5E, F). Additionally, we analyzed the chromosomal locations of genes whose expression differed between males and females. Our findings indicate that genes overexpressed in male patients are primarily located on the Y chromosome. However, for female patients, genes with high expression levels are more prevalent on chromosome 12 than on the X chromosome (Fig. 5F).

### Identification of candidate drugs for AS treatment by machine learning and CMAP analyses

**Fig. 6:**
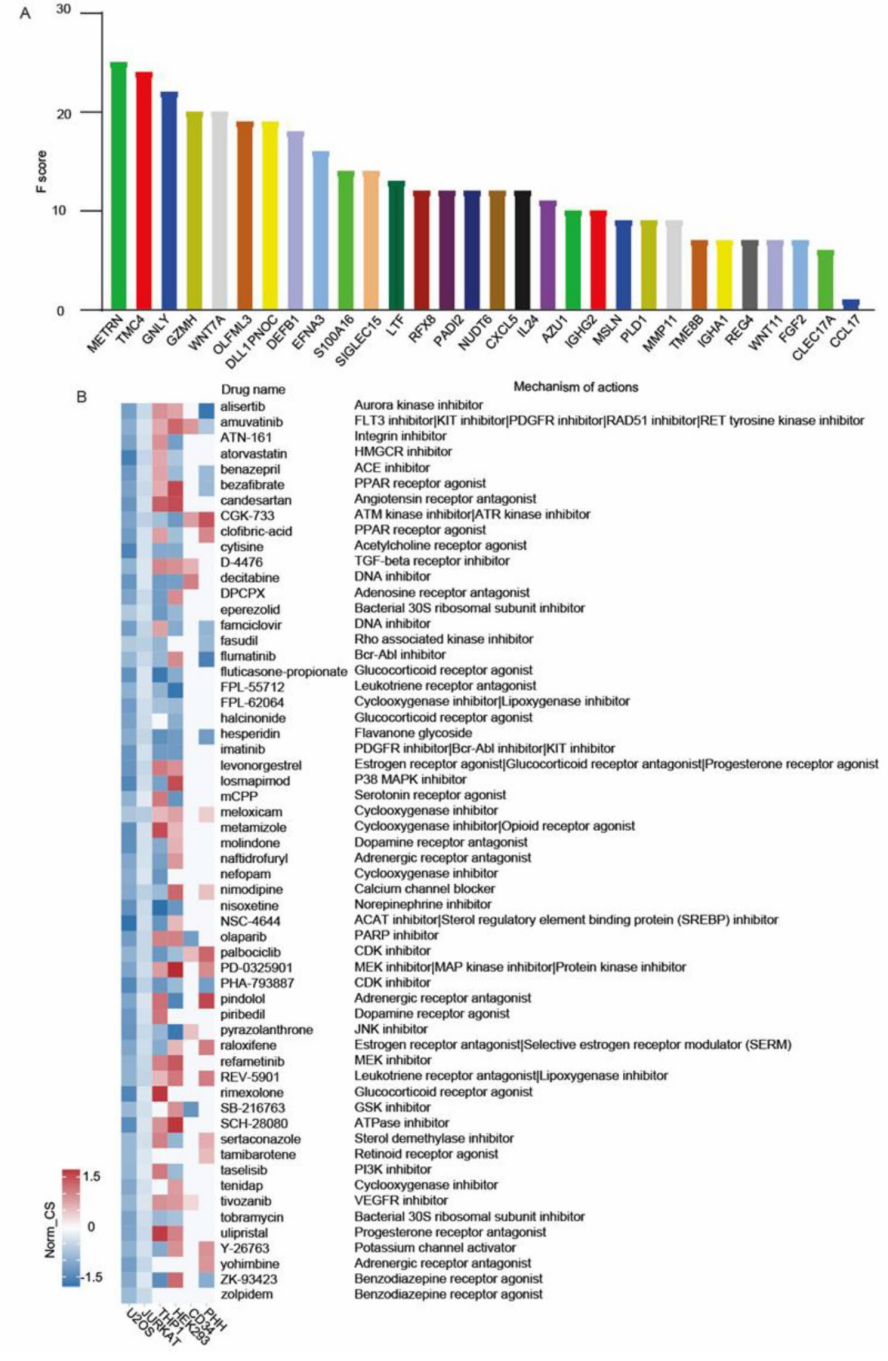
Identification of candidate drugs for ankylosing spondylitis (AS) treatment by machine learning and CMAP analyses. **A** Top 29 features selected using XGBoost and the corresponding variable importance score. Y-axis indicates the importance score, which is the relative number of a variable that is used to distribute the data; X-axis indicates the top 29 weighted variables. **B** CMAP-predicted drugs that may intervene in the occurrence of AS. U-2OS, human osteosarcoma cells. Jurkat cells, human T-cell leukemia cell line. CD34, hematopoietic stem cell line. PHH, primary human hepatocytes. THP1, acute monocytic leukemia cell line. HEK293, human embryonic kidney cell line

Next, we utilized databases GSE221786, GSE73754, GSE25101, GSE181364 and GSE205812 and employed the XGBoost algorithm for machine learning to aid in assessing the impact of the DEGs encoding secreted proteins with elevated levels in the male AS patient on disease onset (Fig. 6A). Specifically, genes such as CXCL5, MMP11 and CCL17 have been reported to promote inflammation [29–31]. In addition, other DEGs, such as WNT7A, WNT11, and FGF2, have been documented to enhance the osteogenic differentiation of mesenchymal stem cells [32–34], a process that is central to the pathogenesis of new bone formation in AS.

We obtained scoring results for the secreted proteins. We also used the CMAP database to search for medications that might affect the activities of these secreted proteins, leading to the identification of potential therapeutic interventions (Fig. 6B). The information derived from the CMAP analysis was also used to identify the progress of candidate drugs through clinical trials and to determine their likely mechanisms of action, providing a reference for the further development of treatments (Fig. 6B). In male patients with AS, we have observed high expression levels of CXCL5, along with enhanced oxidative phosphorylation in IL-17 secreting cells. Previous literature has indicated that statins can attenuate the pro-inflammatory effects of CXCL5 and reduce the production of reactive oxygen species (ROS) resulting from oxidative phosphorylation [35–37]. This could be a reason for the therapeutic efficacy of statins in treating AS. Additionally, there have been reports that ACE inhibitors can suppress the production of CXCL5 and reactive ROS [38, 39]. Therefore, the use of ACE inhibitors, such as benazepril, may help alleviate the inflammatory of AS patients. Hesperidin, as a flavanone glycoside, minimized ROS intracellularly and can reduce the destruction of articular cartilage in osteoarthritis [40]. Perhaps in AS patients, it may also help to reduce cartilage damage.

## Discussion

In this study, we investigated the gene expression patterns of IL17-secreting cells from the blood of both male and female AS patients. Our observations revealed that the activation of the TCR signaling pathway was more prominent in female subjects than in male subjects, while mast cells are more highly activated in male patients. This research offers a potential explanation for the increased BASFI score in female AS patients relative to male AS patients.

Recent advances in single-cell sequencing hold great potential for exploring biological systems with unprecedented resolution. Sequencing the genome of individual cells can reveal somatic mutations and allows the investigation of clonal dynamics [41–43]. However, compared to mRNA sequencing, single-cell sequencing is more expensive, resulting in fewer sequencing cases in public databases and unable to be analyzed through machine learning. Proteomics involves the applications of technologies for the indentification and quantification of overall proteins present content of a cell, tissue or an organism [44]. The proteomic analysis results of AS lesion can be integrated with the sequencing results of peripheral serum to more accurately identify the proteins responsible for local pathogenesis. However, due to the insufficiency of proteomic sequencing data from the AS lesion, we have been unable to combine these two datasets.

Moreover, our analysis extended to the differential activation of immune signaling pathways between Sexs, finding transcriptomic evidence of a notably higher expression of TNF in female patients relative to male patients. This heightened activation, however, does not correlate with the efficacy of TNF inhibitors, which is less pronounced in women. We postulate that this discrepancy may be due to the involvement of additional pathways in ankylosing spondylitis, including the IL-17 pathway and the Wnt pathway, as well as potentially undiscovered pathways. The current clinical paradigm of uniform TNF inhibitor dosages [45–47] for both sexes may not be optimal, as it is conceivable that women might benefit from higher dosages of TNF inhibitors to achieve therapeutic parity with men. Some researchers have found that MTA3 could activate Wnt signaling pathway [48]. Therefore, MTA3 signaling pathways that are more highly activated in male patients may contribute to ligament osteogenesis by activating Wnt signaling pathway.

Additionally, our exploration into the metabolic profiles of these cells uncovered that genes involved in oxidative phosphorylation pathways and lipid oxidation processes were predominantly upregulated in immune cells from male patients as opposed to female patients. The metabolic patterns of T cells help to determine their functionality [49–51]. Metabolism within these cells affects their proliferation, differentiation and overall immune response. Activated effector T cells tend to exhibit augmented anabolic metabolic pathways, such as aerobic glycolysis, while memory T cells tend to more strongly engage catabolic pathways, like fatty acid oxidation [49]. Moreover, select lipids act as metabolic regulators, intertwining environmental signals with cellular signaling pathways to influence T cell biology [51]. In this study, we also uncovered transcriptomic evidence that in male patients, IL-17-secreting cells likely exhibit an increase in both aerobic glycolysis and fatty acid oxidation. Although classical activation pathways appear weaker in male patients as compared to female patients, their state of high-energy metabolism suggests a higher level of cellular activation in male subjects. This indicates that there may exist undiscovered T cell activation pathways that underlie AS.

This study also uncovered some previously unrecognized genes that may be related to AS. For example, transcripts coding for METRN, also known as meteorin, were found to be highly expressed in IL-17-secreting cells in male AS patients and to have an impact on disease occurrence. This protein has not previously been associated directly with AS, although it is known to serve as an important promoter of the formation of axonal networks during neurogenesis. In addition, METRNL has been reported to attenuate lipid-induced inflammation and insulin resistance via AMPK- or PPARδ-dependent pathways in skeletal muscle of mice [52]. Other such proteins that we uncovered include TMC4 (transmembrane channel-like protein 4), which is predicted to enable mechanosensitive ion channel activity and to be involved in ion transmembrane transport, although further research into its biological roles is required. GNLY (granulysin) is a member of the saposin-like protein family and is located in T cell cytotoxic granules, which are released upon antigen stimulation; no other studies have reported connections of its expression with AS. WNT7A promotes the osteogenic differentiation of human mesenchymal stem cells [32], which is considered to be a mechanism driving ligament osteogenesis in AS. However, there are currently no reports on its mechanism of action in AS. We propose that WNT7A might warrant additional research into the mechanism of ectopic osteogenesis in AS.

Our research using CMAP identified several interesting drugs that deserve further exploration in the context of AS. Alisertib, an aurora kinase inhibitor, promotes apoptosis and autophagy in human osteosarcoma U-2 OS and MG-63 cells by activating mitochondrial pathways and inhibiting the p38 MAPK/PI3K/Akt/mTOR signaling pathway [53]. While no studies have investigated the use of this drug in the treatment of AS, we propose that it may reduce ectopic bone formation by promoting apoptosis of local osteoblasts. Atorvastatin, an HMG-CoA reductase inhibitor, is used as a lipid-lowering medication for the treatment of myocardial infarction. It has also been shown, however, to alleviate bone loss and aortic valve atherosclerosis in LDLR mice [54], suggesting that it might also reduce the occurrence of ectopic bone formation in AS.

The limitations of this article lie in the fact that we have not effectively integrated the metabolic profiles and corresponding activation of signaling pathways observed in males and females with the analysis of potentially therapeutic drugs for AS. Given that the CMAP data is predicated on the relationship between secretory proteins and the corresponding cellular expression profiles modulated by drugs, this screening method has the potential to identify candidate drugs that may currently be unknown in their capacity to intervene with the action of secretory proteins. The mechanisms of action of these drugs necessitate further experimental validation and cannot be ascertained solely through the review of existing literature. Additionally, the sum of the proportions of the recognized cell types does not amount to 100%. Previous studies have also reported the isolation of mast cell precursors from PBMCs [55–57]. It is plausible that the presence of mast cell progenitors within PBMCs may have contributed to unrecognized cell type and the enrichment of pathways associated with mast cell activation. Furthermore, incorporating single-cell sequencing data from peripheral blood could have more precisely identified the cell types responsible for the secretion of pathogenic proteins. Regrettably, we were unable to find suitable data for such an analysis.

This study analyzed the sequencing data of blood samples from AS patients and inferred possible factors for disease differences between male and female AS patients. However, further analysis is required to identify mechanisms to explain these sex-based differences. In addition, further in vitro and in vivo research is required to confirm our conclusions and to more deeply investigate the associated factors.

### Electronic supplementary material

Below is the link to the electronic supplementary material.

Supplementary Material 1

Supplementary Material 2

Supplementary Material 3

Supplementary Material 4

Supplementary Material 5

## Data Availability

The datasets analysed during the present study are available in the GEO repository. The collated data can also be found in Supplementary Document 1.
